# Extracellular Vesicles Deliver Host and Virus RNA and Regulate Innate Immune Response

**DOI:** 10.3390/ijms18030666

**Published:** 2017-03-20

**Authors:** Takahisa Kouwaki, Masaaki Okamoto, Hirotake Tsukamoto, Yoshimi Fukushima, Hiroyuki Oshiumi

**Affiliations:** 1 Department of Immunology, Graduate School of Medical Sciences, Faculty of Life Sciences, Kumamoto University, 1-1-1 Honjo, Chuo-ku, Kumamoto 860-8556, Japan; kouwaki@kumamoto-u.ac.jp (T.K.); mokamoto@kumamoto-u.ac.jp (M.O.); htsukamo@kumamoto-u.ac.jp (H.T.); yfuku@kumamoto-u.ac.jp (Y.F.); 2 Japan Science and Technology Agency, PRESTO, 1-1-1 Honjo, Chuo-ku, Kumamoto 860-8556, Japan

**Keywords:** innate immunity, microRNA, virus, extracellular vesicles

## Abstract

The innate immune system plays a crucial role in controlling viral infection. Pattern recognition receptors (PRRs), such as Toll-like receptors and RIG-I-like receptors, sense viral components called pathogen-associated molecular patterns (PAMPs) and trigger signals to induce innate immune responses. Extracellular vesicles (EVs), including exosomes and microvesicles, deliver functional RNA and mediate intercellular communications. Recent studies have revealed that EVs released from virus-infected cells deliver viral RNA to dendritic cells and macrophages, thereby activating PRRs in recipient cells, which results in the expression of type I interferon and pro-inflammatory cytokines. On the other hand, EVs transfer not only viral RNA but also host microRNAs to recipient cells. Recently, infection of hepatocytes with hepatitis B virus (HBV) was shown to affect microRNA levels in EVs released from virus-infected cells, leading to attenuation of host innate immune response. This suggests that the virus utilizes the EVs and host microRNAs to counteract the antiviral innate immune responses. In this review, we summarize recent findings related to the role of EVs in antiviral innate immune responses.

## 1. Introduction

Exosomes are released from multivesicular bodies (MVBs) and deliver functional RNAs, such as mRNA and microRNA (miRNA), to other cells, and thus exosomes mediate intercellular communications [1]. In contrast to exosomes, microvesicles are released from plasma membrane, and it has been shown that microvesicles also deliver functional RNAs and mediate intercellular communications [2]. Recent studies have revealed important roles of these extracellular vesicles (EVs) in controlling antiviral innate immune responses. In the innate immune system, viral RNAs are recognized by pattern recognition receptors (PRRs), such as Toll-like receptors (TLRs) and RIG-I-like receptors (RLRs) [3,4]. In endosomes, viral double-stranded RNA (dsRNA) is recognized by TLR3 [4,5,6,7], whereas single-stranded RNA (ssRNA) is recognized by TLR7 and TLR8 [4,8,9]. In contrast, cytoplasmic viral dsRNAs are sensed by RLRs, such as RIG-I and MDA5, with accessory factors including LGP2 and other cytoplasmic helicases [3,10,11]. Activation of RLRs is regulated by K63-linked polyubiquitination and phosphorylation [12]. For instance, TRIM25 and Riplet ubiquitin ligases mediate the K63-linked polyubiquitination of RIG-I N- and C-terminal regions, which are essential for the production of type I interferon (IFN) [13,14,15]. Other accessory factors are also involved in the activation of RLRs [16,17,18,19]. Activation of these adaptor proteins leads to the production of type I IFN and pro-inflammatory cytokines. PRRs recognize viral DNA as well as viral RNA. TLR9 senses the non-methylated CpG DNA within the endosome [4,20], and cytoplasmic double-stranded DNA (dsDNA) is recognized by a DNA sensor, cyclic-GMP-AMP synthase (cGAS) [21,22,23]. 

**Figure 1:**
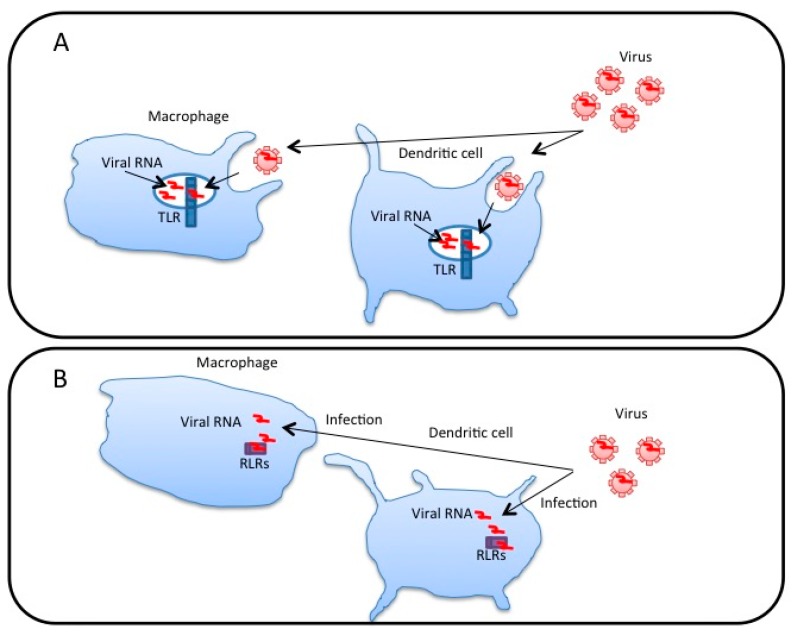

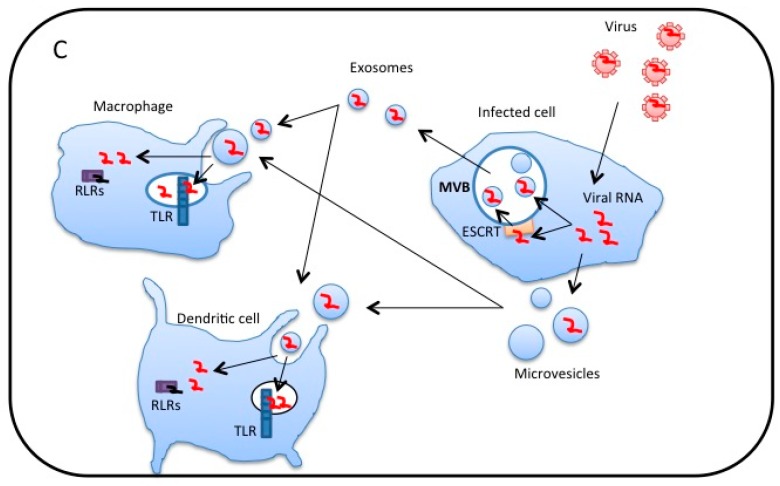
Recognition of viral RNA by pattern recognition receptors. (**A**) Dendritic cells and macrophages internalize virus particles through phagocytosis. Viral RNAs are internalized into endosomes, wherein Toll-like receptors (TLRs) recognize the viral RNA and trigger the signal to induce innate immune responses; (**B**) Some kinds of viruses infect dendritic cells and macrophages. Viral RNA is released into the cytoplasm. Cytoplasmic viral RNA sensors, RIG-I-like receptors (RLRs), detect the viral RNA in the cytoplasm and trigger innate immune responses; (**C**) In virus-infected cells, viral RNAs are sorted into exosomes and microvesicles via endosomal sorting complexes required for transport (ESCRT) or unknown factors. Dendritic cells and macrophages internalize extracellular vesicles (EVs) containing viral RNAs, which are released into endosomes and are recognized by TLRs, resulting in innate immune responses. Viral RNAs released into the cytoplasm were recognized by RLRs.

PRRs are expressed in dendritic cells (DCs) and macrophages that produce large amounts of type I IFNs and pro-inflammatory cytokines [24]. DCs can internalize virus particles by phagocytosis and macropinocytosis. The internalized viral RNAs within endosomes are recognized by TLRs, leading to antiviral innate immune responses [4,25,26] (Figure 1A). TLR7 in plasmacytoid DCs senses influenza A virus RNA [8], whereas TLR3 in conventional DCs recognizes the poliovirus RNA in endosomes [27,28]; although the genome of poliovirus RNA is single-stranded, it forms several short double-stranded regions, which can be recognized by TLR3 [29]. Hepatitis C virus (HCV), dengue virus, vesicular stomatitis virus, Sendai virus, and West Nile virus are recognized by TLR3 and/or TLR7 [30,31,32,33]. Alternatively, viruses invade DCs and macrophages, and the internalized viral components are released into the cytoplasm, wherein RLRs recognize the viral RNA (Figure 1B). RIG-I recognizes the RNA of influenza A virus, vesicular stomatitis virus, Sendai virus, and Japanese encephalitis virus, whereas MDA5 recognizes the viral RNA of encephalomyocarditis virus [34].

Interestingly, recent studies elucidated another route of viral RNA recognition by PRRs. Chisari and colleagues first reported that exosomes released from HCV-infected hepatocytes carry viral RNA and deliver it to plasmacytoid DCs (pDCs) and stimulate TLR7, which results in the production of type I IFN [35] (Figure 1C). Later, we found that conventional DCs (cDCs) and macrophages sense the viral RNAs within EVs released from HCV or hepatitis B virus (HBV)-infected hepatocytes and evoke antivirus innate immune responses via TLRs and RLRs [36,37] (Figure 1C). In this review, we summarize recent findings related to the role of host and viral RNAs within EVs in the antiviral innate immune response [36,37].

## 2. Exosomes and Microvesicles

There are several types of EVs, such as exosomes, microvesicles, and apoptotic bodies. Exosomes are 40–100 nm small vesicles released from MVBs in host cells [1]. They carry functional RNAs such as mRNA and miRNA, and deliver them to recipient cells, which results in intercellular communications [1]. Endosomal sorting complexes required for transport (ESCRT)-machineries, including ESCRT-0, -I, -II, and -III complexes, are essential for exosome biogenesis [38]. The ESCRT-II subunits can bind to RNA, and the ESCRT-machinery plays a crucial role in sorting the cytoplasmic mRNA and miRNA into exosomes [39]. CD9, CD63, and CD81 proteins are enriched in exosomes, and thus, these proteins are considered to be markers for exosomes [40].

In contrast to exosomes, microvesicles, whose diameters are 50 nm–1 μm, are created through direct budding from the plasma membrane. Microvesicles and exosomes are often released concomitantly and thus, it is sometimes difficult to discriminate the roles of exosomes and microvesicles [41]. The lipid bilayer of both exosomes and microvesicles contains phosphatidylserine (like that in apoptotic bodies) [40,42], and a fraction of microvesicles exhibit a diameter similar to that of exosomes. Microvesicles also transfer mRNA and miRNAs to recipient cells in a manner similar to exosomes [2], but the mechanisms underlying the sorting of RNAs to microvesicles remain unclear.

## 3. Extracellular Vesicles Released from Virus-Infected Cells Transfer Viral RNA to DCs and Macrophages

CD81 is a tetraspanin membrane protein and is enriched in exosomes [43]. HCV proteins are known to associate with CD81 for HCV infection of hepatocytes [44]. Therefore, it was expected that HCV would associate with exosomes via CD81. Indeed, it was reported that HCV protein and RNA were associated with exosomes that were captured from the plasma of HCV-infected patients [45]. However, Dreux et al. reported that HCV RNA was packaged within exosomes that were released from HCV-infected hepatocytes [35]. They also showed that exosomes carrying HCV RNA activate pDCs, resulting in type I IFN production [35]. Moreover, knockdown of ESCRT-I or -III complex components in HCV-infected cells attenuated the exosome-mediated type I IFN production by pDCs [35]. These observations indicate that HCV RNA packaged within exosomes was delivered to pDCs, which recognized the HCV RNA via TLR7, leading to type I IFN production. Later, we observed that EVs released from hepatocytes with HCV replicons delivered viral RNA to CD8α^+^ DCs, a subset of cDCs, leading to TLR3-mediated type I and type III IFN production [37]. 

EV-mediated transfer of HBV RNA has also been reported. HBV is a hepadnavirus and is a major cause of hepatocellular carcinoma. Recently, we found that EVs released from HBV-infected hepatocytes contain viral RNA, which is transferred to macrophages [36], resulting in the expression of NKG2D ligands, such as ULBP1 and ULBP2. The expression of NKG2D ligands in macrophages is known to induce NK cell-mediated IFN-γ production [46,47], and we observed that intravenous infection with HBV induced hepatic IFN-γ expression in an animal model [36]. 

The ability to transfer viral RNA to DCs and macrophages via EVs is not specific to vesicles released from hepatocytes. Epstein-Barr virus (EBV) has a strong B cell tropism and has evolved a latency strategy to avoid immune detection. Exosomes released from EBV-infected lymphoblastoid cell lines activate the innate immune response in primary DCs [48]. An EBV transcript, EBER1 RNA with 5′ triphosphate, was transferred to DCs by exosomes, thereby inducing interferon inducible gene expression in DCs [48]. 

Packaging of viral RNA within exosomes was also detected in other studies. The exosomes released from Vero cells infected with Rift Valley Fever Virus also contained viral genomic RNA, and their components were delivered to recipient cells [49]. Moreover, HIV-infected T lymphocytes and macrophages constitutively produce exosomes containing high amounts of trans-activation response element (TAR) RNA, a viral transcript, which was delivered to macrophages and induced the production of pro-inflammatory cytokines in macrophages [50]. An accumulating body of evidence demonstrates that EVs released from virus-infected cells transfer viral RNAs to recipient cells including DCs and macrophages, thereby inducing innate immune responses.

## 4. Roles of MicroRNAs within Exosomes for Regulation of Innate Immune Responses

It has been reported that miRNAs within exosomes regulate innate immune responses. Stimulation with lipopolysaccharide (LPS), which is a ligand of TLR4, increases miR-155 expression levels [51]. Exosomes containing miR-155 are released from bone-marrow-derived DCs (BM-DCs) and are transferred to recipient BM-DCs [52]. The transferred miR-155 increased the inflammatory response in recipient BM-DCs [52]. 

**Figure 2:**
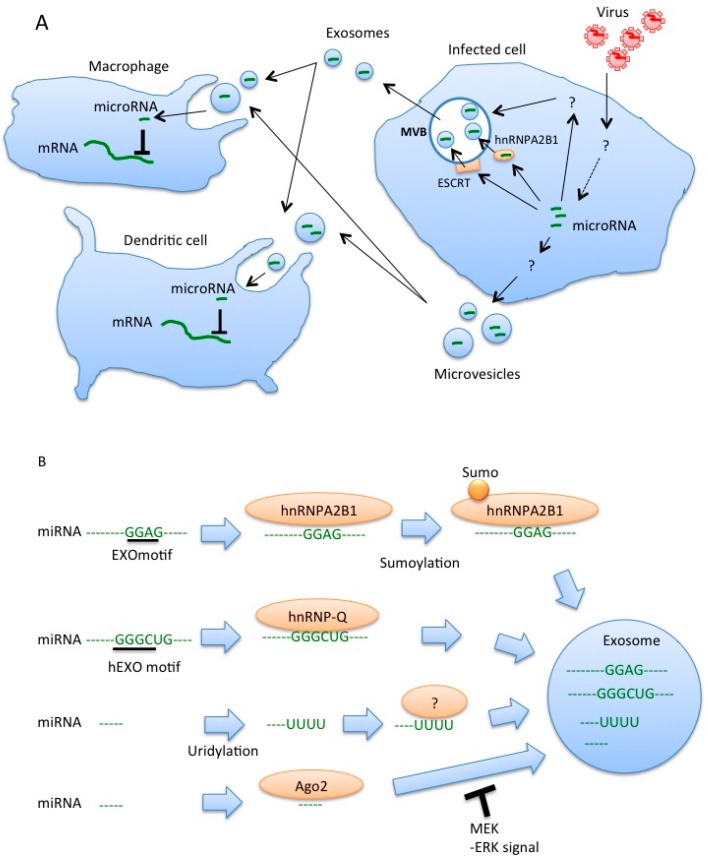
MicroRNAs within the extracellular vesicles regulate the innate immune response. (**A**) miRNAs are sorted into exosomes via the ESCRT complex, hnRNPA2B1, and other proteins. It has been reported that viral infection affects the microRNA levels in EVs. Dendritic cells and macrophages uptake miRNA-containing EVs. Internalized miRNAs are released into the cytoplasm and reduce the target mRNA expression or translation; (**B**) There are several pathways by which cytoplasmic miRNAs are sorted into exosomes. hnRNPs and Ago2 proteins are involved in the miRNA sorting, and post-translational modification of miRNA or proteins associating with miRNAs affect the sorting processes (see main text). MVB, multivesicular bodies. The proteins required for sorting of uridylated miRNA remain unclear.

Although the innate immune system is the first line of defense against virus infection, many viruses have evolved to escape the host innate immune response in order to infect host cells [12]. Recently, we found that HBV attenuates the innate immune response via the miRNA within exosomes (Figure 2). The X protein of HBV plays a key role in the molecular pathogenesis of HBV-related hepatocellular carcinoma (HCC) [53,54]. Expression of the X protein of HBV induces miR-21 expression in HCC cell lines [55]. IL-12 is a heterodimeric cytokine, which consists of p35 and p40 subunits, and is produced by DCs and macrophages [56]. The miR-21 targets the 3′ UTR of IL-12p35, resulting in the reduction of the target protein levels [57]. Interestingly, we found that the expression of the HBV proteins in HCC cell lines increased the exosomal miR-21 levels [36]. Moreover, we found that exosomal miR-29 level was also increased by the expression of HBV proteins in HCC cell lines [36]. miR-29 targets IL-12p40 [58], which is another subunit of IL-12. Therefore, the expression of HBV proteins in HCC cell lines increased exosomal miR21 and miR-29 levels, which target each subunit of IL-12. IL-12 is well known to activate natural killer (NK) cells [59], and NK cells play a crucial role in suppressing HBV proliferation [60]. When macrophages were incubated with the exosomes released from HBV-infected cells and were then activated by TLR ligands, IL-12 production was reduced by exosomes released from HBV-infected cells compared to those released from mock-infected cells. These observations indicate that HBV proteins increase the exosomal miR-21 and miR-29 levels to attenuate the IL-12 production from macrophages and to counteract host innate immune responses.

## 5. Sorting of miRNA into Exosomes

ESCRT machineries are reported to be capable of sorting HCV RNA into exosomes, whereas the mechanisms of sorting of other virus RNAs into exosomes have not been elucidated. In contrast, recent studies have shed light on the mechanisms of miRNA sorting into exosomes (Figure 2). The hnRNPA2B1 protein is a ubiquitously expressed RNA-binding protein, and binds to a short motif within miRNA, which is called the EXOmotifs [61]. miRNAs with EXOmotifs are preferentially sorted into exosomes, and miRNAs without the motif remain in the cytoplasm in primary T lymphocyte [61]. In addition, sumoylation of hnRNPA2B1 promotes the sorting of miRNAs with EXOmotif into exosomes [61]. In hepatocytes, miRNAs with another motif, hEXO motif, were sorted into exosomes via a hnRNP, hnRNP-Q, also called SYNCRIP [62]. Another study has shown that the Y-box protein 1 is involved in miR-223 sorting to exosomes in a cell-free system [63]. Post-translational modification is also involved in miRNA sorting. For example, uridylation of 3′ ends of miRNAs promotes their sorting to exosomes in B cells [64]. 

The Ago2 protein binds to miRNA, and the Ago2-miRNA complexes are released from cells as free proteins [65]; however, there are studies showing that Ago2 is packaged into exosomes along with miRNA [66,67]. Interestingly, it was shown that MEK-ERK activation inhibits the sorting of Ago2 and Ago2-miRNA complex into exosomes [68]. Activation of TLRs affects ERK signaling [69]. Considering that HBV infection is detected by TLRs [36], it is possible that the increase in miR-21 and miR-29a levels in exosomes after HBV infection could be regulated by the activation of TLRs. 

## 6. Uptake of Exosomes

Exosomes are efficiently internalized through phagocytosis [70], and macrophages and DCs internalize exosomes very efficiently [70,71]. Therefore, host and viral RNA within exosomes are expected to be efficiently transferred to DCs and macrophages compared to other types of cells. The proteins involved in the uptake of exosomes by DCs and macrophages have been reported. Treatment with cytochalasin D reduced exosome uptake [71], suggesting the requirement of actin polymerization. In addition, blocking antibodies against α_v_ (CD51) and β_3_ (CD61) integrins, LFA-1 (CD11a), CD54 (ICAM-1), CD9, and CD81 reduced the uptake of exosomes [1,71]. Moreover, the soluble analog of phosphatidylserine also reduced exosome uptake [71], suggesting that the phosphatidyl serine on exosomes is recognized by recipient DCs. The integrin α_v_β_3_ is expressed in macrophages and binds MFG-E8, which is a soluble factor associating with phosphatidyl serine [72,73]. CD9 and CD81 are enriched in the membrane of the exosomes [38], and bind the α_v_β_3_ integrin [74]. CD54 (ICAM-1) is a ligand of LFA-1 (CD11a) [75]. LFA-1 (CD11a) is expressed in DCs, and it has been shown that activated T cells bind to DC-derived EVs through LFA-1 [76]. In addition, the expression of α4 integrin promotes the uptakes of EVs by lymph node stromal cells [77], and macrophages require galectin-5 expression on EVs released from erythrocytes [78]. Considering that blocking these proteins with antibodies only partially reduced the uptake, it is expected that the recipient cells internalize EVs through several redundant uptake pathways. 

The fusion of the exosome membrane with the membrane of recipient dendritic cells or cancer cells has been demonstrated using assays with the lipophilic dye R18, which has been used to monitor the fusion of enveloped viruses and lipid vesicles. The R18 dye is a self-quenching fluorescent lipid probe at its high concentration. When exosomes labeled with self-quenched R18 were incubated with recipient DCs, the fluorescence immediately increased, suggesting the fusion of the exosome membrane with the recipient DC membrane [79]. This exosome membrane fusion with recipient cells was also observed in the case of cancer cells [1]. 

The release of the luminal content of exosomes into the cytosol of DCs has also been reported. When the exosomes containing the luciferin were incubated with DCs expressing luciferase, emission of light was observed at 10 min after incubation [79]. Moreover, miR-148a and miR-451 within the exosomes were internalized into the recipient DCs and they down-regulated their target [79]. 

These facts are consistent with the notion that miRNAs within EVs released by viral-infected cells are transferred into the cytoplasm of recipient cells and that they regulate the innate immune response. In the case of HBV infection, the expression of HBV proteins increased the miR-21 and miR-29 levels in EVs, which are transferred into macrophages [36], whereas the role of cell surface proteins involved in the uptake of EVs has not been fully elucidated. Further studies are required to uncover the underlying mechanisms. 

## 7. Perspectives

Recent studies have elucidated the crucial roles of RNA within EVs on antiviral innate immune responses, as described above. Therefore, several researchers have tried to use the miRNA within EVs as new biomarkers or to utilize EVs with RNAs as novel drugs. Several studies have already shown that exosomal miRNAs can be used as biomarkers of cancer diagnosis [80,81]. Considering that viral infection changes the miRNA levels in EVs released form virus-infected cells, the miRNA levels in EVs can be used as a biomarker for the diagnosis of infection. EVs are enriched in human serum, and serum exosomal miR-122 is significantly increased by liver injury and inflammation caused by virus infection, and thus it is postulated that miR-122 serves as a biomarker of liver damage and inflammation [82]. 

The loop-mediated isothermal amplification (LAMP) method can detect specific DNA or RNA within samples by incubating the samples for 15–50 min at 65 °C. The LAMP method does not require a thermal cycler, but only requires a simple incubator [83]. miRNAs can be detected by a modified LAMP method [84]. Because EVs are enriched in human blood or human serum samples, it is expected that the miRNAs within serum EVs can be easily detected by using modified LAMP methods and can also be used for the diagnosis of infectious disease in a short period and at low cost in hospitals or in epidemiological studies. 

In addition, because of the effect of miRNAs within EVs on DCs and macrophages, it has been postulated that EVs with miRNA can be used for the treatment of infectious diseases or for developing a vaccine. For instance, miR-21 and miR-29 reduce the production of IL-12, which is known to promote Th1 differentiation, and thus it is expected that EVs with miR-21 and miR-29 could modulate Th1 versus Th2 response patterns and activate cytotoxic T lymphocytes (CTL) and NK cells. This might be useful to attenuate cytotoxicity against HBV- or HCV-infected hepatocytes by CTL or NK cells and to increase the production of neutralizing antibodies by Th2 response. Moreover, miR-155 is shown to augment innate immune responses, and thus EVs with miR-155 are expected to augment the adjuvant effects of vaccines, thereby improving the efficacy of vaccines. Further studies of EV-mediated immune regulation will help to establish new strategies for the treatment and prevention of infectious disease using EVs with miRNA.
